# The Influence of Capsaicin on the Integrity of Microvascular Endothelial Cell Monolayers

**DOI:** 10.3390/ijms20010122

**Published:** 2018-12-30

**Authors:** Mathias Kaiser, Malgorzata Burek, Stefan Britz, Frauke Lankamp, Steffi Ketelhut, Björn Kemper, Carola Förster, Christian Gorzelanny, Francisco M. Goycoolea

**Affiliations:** 1 Max Delbrück Center for Molecular Medicine, Robert-Rössle-Straße 10, 13125 Berlin, Germany; Mathias.Kaiser@mdc-berlin.de; 2 Institute of Plant Biology and Biotechnology (IBBP), Westfälische Wilhelms-Universität Münster, Schlossgarten 3, 48149 Münster, Germany; stefan.bulla@gmx.at (S.B.); f_lank01@uni-muenster.de (F.L.); 3 Deparment of Anaesthesia and Critical Care, University of Würzburg, Oberdürrbacher Straße 6, 97080 Würzburg, Germany; Burek_M@ukw.de (M.B.); foerster_c@ukw.de (C.F.); 4 Biomedical Technology Center of the Medical Faculty, Westfälische Wilhelms-Universität Münster Mendelstraße 17, 48149 Münster, Germany; ketelhut@uni-muenster.de (S.K.); bkemper@uni-muenster.de (B.K.); 5 Department of Dermatology and Venerology, University Medical Center Hamburg-Eppendorf, 20246 Hamburg, Germany; c.gorzelanny@uke.de; 6 School of Food Science and Nutrition, University of Leeds, Leeds LS2 9JT, UK

**Keywords:** tight junctions, capsaicin, endothelial cells

## Abstract

Microvascular endothelial cells are an essential part of many biological barriers, such as the blood–brain barrier (BBB) and the endothelium of the arteries and veins. A reversible opening strategy to increase the permeability of drugs across the BBB could lead to improved therapies due to enhanced drug bioavailability. Vanilloids, such as capsaicin, are known to reversibly open tight junctions of epithelial and endothelial cells. In this study, we used several in vitro assays with the murine endothelial capillary brain cells (line cEND) as a BBB model to characterize the interaction between capsaicin and endothelial tight junctions.

## 1. Introduction

Capsaicin occurs in chili peppers as the main compound responsible for pungency [1]. Capsaicin is known to ease chronic pain, change body temperature, and reduce obesity [2,3] among other known bioactivities. Recently, it was found that capsaicin is able to reversibly open tight junctions [4], a protein network interconnecting epithelial cell layers [5]. We have previously exploited this effect to enhance the permeability of macromolecules by designing a nanocapsule formulation comprising an oil core coated with chitosan, a pseudo-natural aminopolysaccharide [6,7]. Furthermore, the impact of capsaicin derivatives on epithtlial tight junctions was investigated [8]. In this study, we investigated whether capsaicin is able to reversibly open endothelial cell monolayers. The blood–brain barrier (BBB) is a physical and enzymatic barrier, whose primary function is to preserve brain homeostasis. As a result, the delivery of potential therapeutic agents to the brain is limited, and thus the treatment of many central nervous system (CNS)-related diseases is hampered. [9]. In vivo studies have shown that the use of capsaicin can lead to a breakdown of the BBB [10] and that the permeability of the microvascular system can increase after application [11]. In this study, we investigated the response of murine microvascular endothelial cells to capsaicin and its synthetic analogue, nonivamide, using the cell line cEND as a model [12,13]. To this end, we determined the cytotoxicity of the compounds and used impedance spectroscopy to probe tight junction integrity by measuring the transendothelial electric resistance (TEER). Impedance spectroscopy confirmed the opening of the tight junctions, with a decrease in TEER in a dose-dependent manner, which reversed upon removal of capsaicin. A decrease in TEER correlated with a decrease in claudin-5 expression in cEND cells, as shown by western blot and qPCR. Changes in cell morphology were examined using structural illumination fluorescence microscopy (SIFM) and digital holographic microscopy (DHM). The study revealed capsaicin-induced changes in the endothelial cells that affected the actin skeleton as well as the tight junctions by means of the dislocation of claudin 5 proteins for cEND and primary endothelial cells.

## 2. Results

### 2.1. Quantified Impact of Vanilloids on the Viability of cEND Cells Using MTT Assays

**Figure 1:**
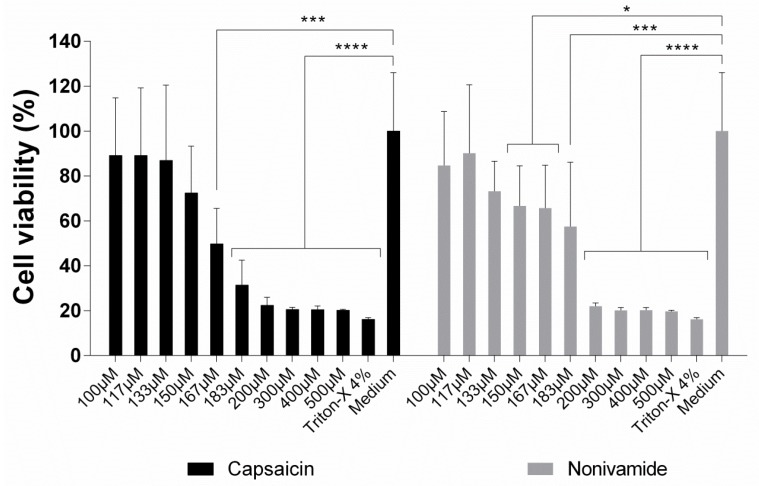
Toxicity of capsaicin and nonivamide against cEND cells in 96-well plates determined using the 3-(4,5-dimethylthiazol-2-yl)-2,5-diphenyltetrazolium bromide (MTT) assay. Relative cell viability following treatment with the compounds at increasing concentrations. For all experiments, cells were incubated for 3 h. Mean values ± SD. Statistical test: Kruskal–Wallis test (*n* = 3, * *p* < 0.05, *** *p* < 0.001, **** *p* < 0.0001).

To assess the metabolic competence of cells upon treatment with capsaicin or nonivamide, we used the 3-(4,5-dimethylthiazol-2-yl)-2,5-diphenyltetrazolium bromide (MTT) assay. Increasing concentrations of both compounds were evaluated over an incubation period of 3 h, and the results are presented in Figure 1. In the case of capsaicin, the viability of the cells started to decrease at a concentration of 150 µM. At a concentration of 167 µM, the effect was statistically significant in comparison to the negative control (*p* < 0.001, Kruskal–Wallis test). Higher concentrations led to a further loss of cell viability until reaching the level of the positive control. In the case of nonivamide, the decrease was slightly more pronounced, leading to a statistically significant decrease in viability at a concentration of 150 µM (*p* < 0.05, Kruskal–Wallis test). It is worth noting that there was a steep decrease in cell viability between 183 µM (*p* < 0.001, Kruskal–Wallis test) and 200 µM (*p* < 0.0001, Kruskal–Wallis test) for nonivamide, which was characteristic of an “all-or-none” response.

We had tested the influence of nonivamide on epithelial systems in a previous study [8]. The cytotoxicity and changes in tight junction integrity was almost identical to capsaicin itself. As the cytotoxic response of nonivamide was also similar to capsaicin in this study, we decided not to further pursue the study of this compound.

### 2.2. cEND Cells Showed Reversible Tight Junction Opening after Temporal Vanilloid Treatment

**Figure 2:**
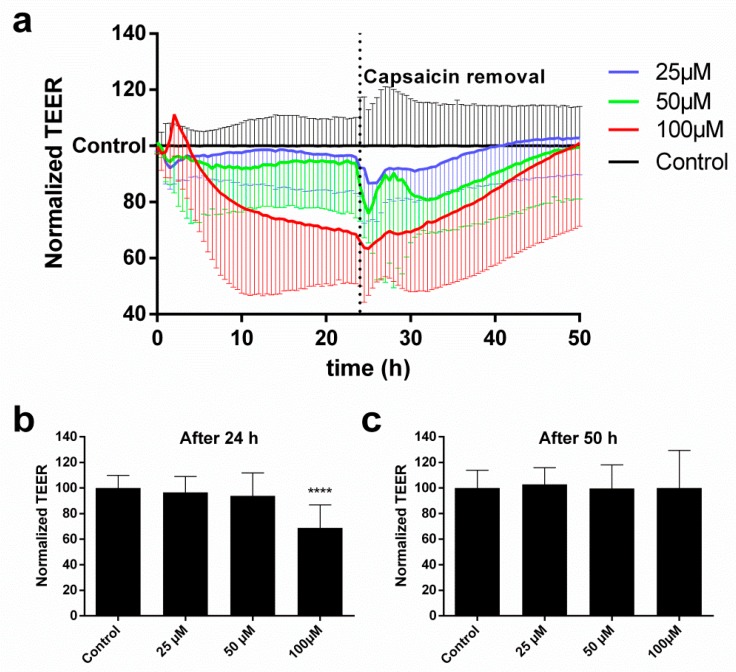
Impedance spectroscopy measurements of transendothelial electric resistance (TEER). (**a**) The normalized TEER relative to the control shown over time for increasing concentrations of capsaicin. The TEER values at the time points of (**b**) 24 h and (**c**) 50 h shown as bar plots. Data are mean values ± SD (*n* = 3, **** *p* < 0.0001).

To study the integrity of the endothelial tight junctions, we conducted impedance spectroscopy measurements to determine the evolution of TEER at increasing capsaicin concentrations. Figure 2a shows the time course of the relative TEER response. Capsaicin was applied on the cells for 24 h and then subsequently removed to study the recovery of the cells up to a total experiment time of 50 h. It is worth noting that the effect of capsaicin on the tight junctions was dose-dependent. While the overall decrease in TEER was not very pronounced for 25 and 50 µM, a monotonic decrease was visible for 100 µM. Close inspection of the 100 µM trace showed that there was an initial increase in TEER that peaked at ~2 h before the onset of the subsequent decrease. After removal of capsaicin and replacement with supplemented cell culture medium, the TEER response of the cells dropped further for a short time, presumably as a consequence of medium change, which recovered within 2 h. Beyond this point, the TEER of the cells recovered to the initial value for all investigated concentrations. Figure 2b shows the endpoint TEER average values for all treatments after 24 h. In the case of 100 µM capsaicin, the decrease in TEER was statistically significant (*p* < 0.0001, Kruskal–Wallis test). However, after 50 h, the TEER of the cells under this treatment recovered to its initial value and showed no significant differences with the control; the cells treated with lower concentrations also did not show any significant differences (Figure 2c).

Impedance spectroscopy measurements allowed us to register the capacitance signal (CCL) concomitantly to the TEER one. The CCL is a robust indicator of morphological changes and cell detachment and correlates closely with other assays of cell viability [6,14,15]. Our data showed that, for the investigated concentrations (25, 50, and 100 µM), the CCL did not increase, indicating that the cells had maintained their viability.

### 2.3. Capsaicin Decreased Expression Levels of Claudin 5 and Induced Dephosphorylation of Cofilin

**Figure 3:**
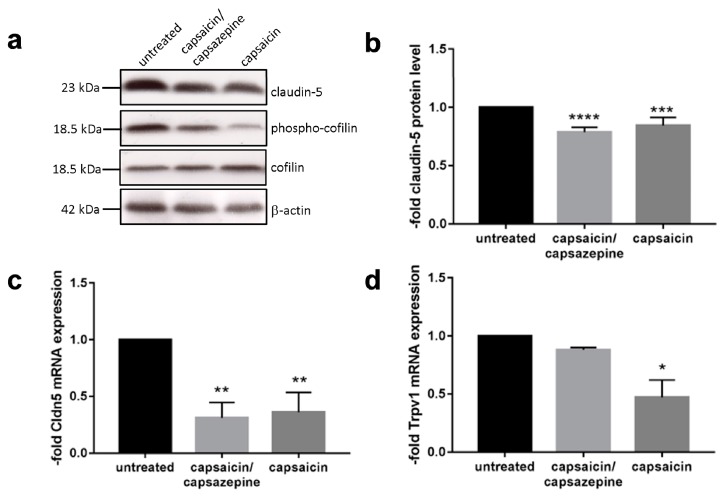
(**a**) Western blot after 12 h of capsaicin exposure. (**b**) Quantification of claudin 5 from western blot. (**c**) Real-time q-PCR of claudin 5 after 12 h of capsaicin exposure. (**d**) Real-time q-PCR of Trpv1 after 12 h of capsaicin exposure. Data was normalized to the untreated control, which was set as 1. Data are mean values ± SD (*n* = 3, **** *p* < 0.0001, *** *p*< 0.0004, ** *p* < 0.01, * *p* < 0.05).

To see if the abundance of claudin 5 is affected by the presence of capsaicin, we looked at its expression on RNA and protein levels. The western blot and the analysis of claudin 5 in capsaicin-treated cells are shown in Figure 3a,b. It can be seen that the claudin 5 protein level decreased by around 20% after 12 h of capsaicin exposure (*p* < 0.0004, one-way ANOVA with Dunnett’s multiple comparisons test). More drastically, this effect was observed on the RNA level, with qPCR experiments showing that the mRNA level of claudin 5 decreased more than 50% after 12 h (Figure 3c) (*p* < 0.01, one-way ANOVA with Dunnett’s multiple comparisons test). Interestingly, this effect was not able to be negated by administration of the capsaicin inhibitor capsazepine, which competitively binds to the capsaicin receptor Trpv1. Nevertheless, Trpv1 was expressed by the cEND cells (Figure 3d). For this receptor, it was visible that upon capsaicin exposure, the receptor was downregulated by 50% (*p* < 0.05, one-way ANOVA with Dunnett’s multiple comparisons test). As expected, the coadministration of capsazepine prevented the downregulation of Trpv1, showing that the inhibitor was functional for its target. Cofilin is one of the actin-depolymerizing factors. Dephosphorylation activates its actin-depolymerizing functions, which is correlated with a decrease in tight junction proteins of epithelial cells [16]. Thus, we checked the cofilin phosphorylation status in our system (Figure 3a). In untreated cEND cells, cofilin was highly phosphorylated, while treatment with capsaicin led to dephosporylation and activation of cofilin.

### 2.4. Fluorescence and Digital Holographic Microscopy Revealed Morphological Changes of Endothelial Cells

The morphological changes of the cells after capsaicin addition were investigated by digital holographic microscopy (DHM) (Supplemental Figure S1). The addition of capsaicin induced the formation of vesicles in the cells, which were not evident in the control treatment. Apart from this finding, no other changes in morphology were detected.

**Figure 4:**
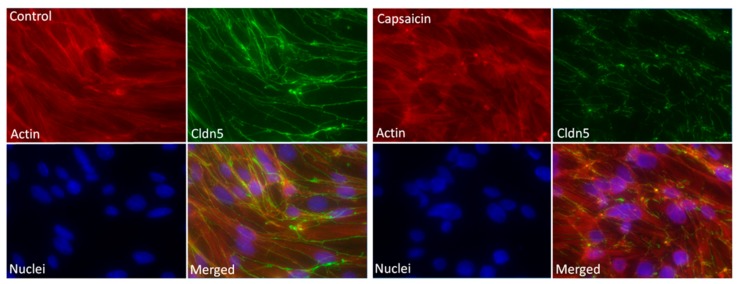
Effect of capsaicin on claudin 5. Structured illumination fluorescence microscopy (SIFM) images of cEND cells treated with capsaicin. Cells remained untreated or were treated with 100 µM capsaicin for 12 h. Nuclei were stained with 2-(4-amidinophenyl)-6-indolecarbamidine dihydrochloride (DAPI; blue), claudin 5 was stained using specific antibodies (green), and actin was stained with tetramethylrhodamine (TRITC)–phalloidin (red), magnification 400×.

Changes of the protein arrangement within the tight junctions, as well as the structural changes of the actin skeleton induced by capsaicin, were imaged using structured illumination fluorescence microscopy (SIFM). Figure 4 shows two panels of SIFM micrographs with cells used as control and treated with 100 µM of capsaicin for 12 h. The staining of actin and claudin 5 was continuously and sharply located on the endothelial contour of the control cells. In contrast, a meticulous inspection showed that claudin 5 rearranged after capsaicin exposure, which was evidenced by diffused, discontinuous, and disrupted staining zones. Furthermore, in the case of the actin skeleton, while the control showed a very organized structure in the capsaicin-treated cells, a dislocation of the proteins was evident. The merged images furthermore substantiated the impression of this overall loss of structural arrangement. These experiments were also carried out for primary mouse brain microvascular endothelial cells (Supplemental Figure S2). In this case, a loss of structure for actin fragments as well as a weakening of the claudin 5 signal was also visible.

To investigate the dislocation of proteins further, we investigated the delocalization events of zonula occludens 1 (ZO-1), which is bound to the claudin and occludin protein families. Microscopical analysis showed that dislocations were visible after the addition of capsaicin to cEND cells (Supplemental Figure S3).

## 3. Discussion

In this study, we investigated the effect of capsaicin on the tight junction integrity of endothelial cells. To this end, we used a combination of biophysical and biological assays as well as microscopy to study the cell line cEND as an in vitro model of the capillary brain endothelium. Firstly, we investigated the cytotoxic response of the cells on capsaicin and nonivamide, its synthetic analogue. Cell viability studies after the MTT assay revealed that both substances caused a continuous decrease in metabolic activity starting at a concentration of around 150 µM when incubated for 3 h. This value was lower compared to the cytotoxic dose that was found in previous studies by our own group, where Madin-Darby Canine Kidney (MDCK) [6] and human colon carcinoma (Caco-2) cells [17] showed a reduction in cell viability at a concentration of 300 µM in the same assay. Impedance spectroscopy measurements of CCL were in good agreement with the MTT results, showing that there was no indication of significant reduction in cell viability for an incubation time of 24 h.

TEER experiments showed that capsaicin was able to disrupt tight junctions of endothelial cells in a dose-dependent manner. However, it was only able to induce a statistically significant effect at a dose of 100 µM. Compared to epithelial cells, endothelial cells were not able to recover their TEER without removal of capsaicin-containing medium [6]. Nevertheless, after removal, the TEER recovered fully at all applied concentrations after about 30 h. Yet another difference to endothelial cells was the shape of the TEER curve. While a widened W shape was visible for epithelial cells [6], only a continuous decrease was observed in the case of endothelial cells.

Western blot and qPCR experiments showed that the level of claudin 5 decreased upon capsaicin exposure. Interestingly, the inhibitor capsazepine was not able to prevent the effect even though Trpv1 was expressed by the cells. However, the expression level of Trpv1 was rather low in cEND cells (Ct values in qPCR between 36 and 38, while strongly expressed claudin 5 showed *C*t values of 19–22). This finding indicated that capsaicin must influence the level of claudin 5 via another pathway. We were able to show that cofilin was activated via dephosphorylation, which is known as an actin-depolymerizing factor. Moreover, in a study of epithelial cells, this factor was responsible for loss of tight junction integrity after capsaicin exposure [16].

We did not find evidence of major changes in the morphology of the cEND cells by DHM upon treatment with capsaicin. In contrast, SIFM imaging did reveal several structural alterations within the cells, particularly a dislocation of claudin 5 proteins in combination with ZO-1. This phenomenon is diagnostic of tight junction network reorganization because ZO-1 serves as the connecting element between the actin skeleton and the transmembrane proteins, such as occludin and the family of claudins. In our study, we observed a redistribution of the actin skeleton, which was consistent with the observed effects after capsaicin treatment on epithelial cells [4,16]. As cofilin was able to depolymerize actin structures, we think that its activation was responsible for the observed reorganization of the actin skeleton and the connected tight junction proteins. Similar results were obtained with primary mouse endothelial cells and in our previous studies with MDCK cells [18]. The staining of other tight junction proteins in future studies could be extremely informative towards the elucidation of the mechanistic underpinning at play. For epithelial cells, it was found that the amount of occludin was downregulated after capsaicin treatment [4]. Further studies to investigate the role of this, as well as other tight junction and cytoskeleton proteins after stimulation with vanilloids in endothelial cells, could lead to gleaning further insight.

In summary, we confirmed that capsaicin is able to reversibly open tight junctions of endothelial cell monolayers in a dose-dependent manner. This makes capsaicin a potential candidate compound for development of drug carriers with the capacity to overcome endothelial cell barriers, such as the BBB. This could lead to advances in tackling diseases like Alzheimer, which call for new strategies to increase the bioavailability of currently used drugs of inherent low permeability (e.g., galantamine). The co-encapsulation of capsaicin and related compounds and such drugs into colloidal “soft” nanoparticles, as well as the influence of this on their permeability across the BBB, will also be an area to address in future studies.

## 4. Materials and Methods

### 4.1. Cell Culture

Brain microvascular endothelial cells (cEND) cells from mouse were kindly donated by the group of Prof. Carola Förster of University of Würzburg. The cells were cultured in 75 cm² flasks using endothelial cell medium (ECM) (CellBiologics; Endothelial Cell Medium + Supplement Kit with VEGF 0.5 mL, heparin 0.5 mL, EGF 0.5 mL, ECGS 0.5 mL, hydrocortisone 0.5 mL, l-Glutamin 5 mL, antibiotic–antimycotic 5 mL, FBS 25 mL before use). Primary mouse endothelial cells were purchased from PeloBiotech (Planegg, Germany) and were cultured as cEND. The cultures were maintained in an incubator at 37 °C with 5% CO_2_ (Sanyo MCO-19AIC, Panasonic Biomedical Sales Europe BV, AZ Etten Leur, Netherlands). For this project, cells from passages 33–43 were used. These experiments were carried out as independent triplicates on different days. After becoming confluent, the cells were washed with 10 mL PBS and detached with 10 mL 0.05% trypsin in EDTA (1×) buffer. After detachment, the trypsin was quenched with 10 mL of ECM. The cell suspension was centrifuged at 1000 rpm for 5 min (Rotina 420 R, Hettich GmbH, Tuttlingen, Germany). The medium was aspirated, and the cell pellet was resuspended in 1 mL ECM. A 10-µL cell suspension was mixed with 90 µL trypan blue, and the cell number was determined with an improved Neubauer chamber before seeding. The cells were propagated by splitting at a ratio of 1:3.

### 4.2. 3-(4,5-Dimethylthiazol-2-yl)-2,5-Diphenyltetrazolium Bromide (MTT) Assay

The cytotoxicity of the compounds was determined using an MTT assay. Briefly, about 10^4^ cells per well in 100 µL of medium were seeded in a 96-well tissue culture plate and allowed to attach for 24 h. Before the experiment, the cells were washed twice with supplement-free ECM before the addition of the sample. The cells were incubated for 3 h. Subsequently, the samples were removed and replaced with 100 µL of supplement-free ECM. An MTT solution in PBS with a concentration of 5 mg/mL of thiazolyl blue tetrazolium bromide was prepared, and 25 µL was added to each well. The medium was again removed after 4 h, and the dye was dissolved in DMSO. The plate was mixed with orbital shaking at 300 rpm for 15 min. The absorbance was measured at *λ* = 570 nm in a microplate reader (Safire, Tecan AG, Salzburg, Austria). Relative viability values were calculated by dividing the individual values by the mean of the control. We used 4% Triton X-100 in PBS as a positive control.

### 4.3. Electrical Impedance Measurements

An automated CellZscope^®^ instrument (nanoAnalytics, Münster, Germany) was used to record the TEER of the cell monolayers. Roughly 1.5 mL of cell culture medium was applied to the basolateral compartment of 12-mm Transwell permeable supports with BD Falcon 0.4-µm pore PET track-etched membrane cell culture inserts (Corning Inc., Corning, NY, USA). The cEND cells were then seeded onto the membrane supports (~10^5^ cells in 500 µL per well) and left to grow for 4 days. Approximately 24 h before the experiment, 1.5 mL of ECM was added to every well, and the cell culture medium of the supports was replaced in the apical compartment with ECM. The supports were moved into the machine and allowed to acclimate. The cell monolayers showed a TEER between 25 and 50 Ω × cm² at the beginning of the experiment. The experiment was initiated by adding 55 µL of capsaicin-containing medium to the apical chamber to yield the desired concentration for the experiment. The resistance was measured continuously for 24 h. The normalized TEER values were calculated using Equation (1):

(1) $$\mathrm{Normalized}\;\mathrm{TEER}=\frac{1}{3}\overset{3}{\underset{j=1}{\sum }}\frac{{\mathrm{Sample}}_{j}}{\left(\frac{1}{3}\sum_{i=1}^{3}{\mathrm{Control}}_{i}\right)}$$

### 4.4. Structured Illumination Fluorescence Microscopy (SIFM)

Cells were seeded on glass slides and allowed to attach for 48 h. The cells were treated with 100 µM capsaicin in medium for 12 h and subsequently washed 2 times with HEPES buffer and fixed with methanol (−20 °C) for 20 min. After washing 3 times with HEPES buffer, unspecific binding was blocked using 2% BSA in incubation buffer (IK) consisting of 0.3% Tween-20, 0.1% BSA in HEPES for 60 min at room temperature. The cells were washed 3 times with IK and incubated with anti-claudin 5 antibody (Thermo Fisher Scientific Inc, Darmstadt, Germany) diluted (1:150) in IK for 4 h at room temperature. Subsequently, the cells were washed 5 times with HEPES buffer and once with IK. As the second antibody, FITC-labeled secondary antibody diluted (1:200) in IK was used, and cells were incubated 1 h at room temperature without light. Cells were simultaneously treated with tetramethylrhodamine (TRITC)-phalloidin (Thermo Fisher Scientific Inc, Darmstadt, Germany) diluted (1:1000) in IK. The cells were then washed 6 times with IK, and the nuclei were stained with 500 ng/mL 2-(4-amidinophenyl)-6-indolecarbamidine dihydrochloride (DAPI; Sigma-Aldrich GmbH, Steinheim, Germany) for 10 min at room temperature. Cells were washed 3 times with IK and 3 times with aqua dest. Coverslips were mounted in DABCO-Mowiol (Sigma-Aldrich GmbH, Steinheim, Germany), dried for 1 h at room temperature, and analyzed by SIFM using an inverted fluorescence microscope (AxioObserver.Z1, Zeiss, Jena, Germany) equipped with a structured illumination module (ApoTome, Jena, Germany).

### 4.5. Western Blot

CEND were seeded on 6-well plates and grown to confluence as described above. The cells were left untreated, were pretreated with 10 µM capsazepine (Sigma) for 30 min, and/or were treated with 100 µM capsaicin for 12 h. Total protein extracts were prepared and the western blot analysis was performed as described previously [19]. Anti-claudin-5 antibody (dilution 1:500; Thermo Fisher Scientific), anti-cofilin (dilution 1:1000, Cell Signaling Technology), anti-phospho-cofilin (Ser3) (dilution 1:1000, Cell Signaling Technology) and anti-β-actin antibody (dilution 1:25.000; Sigma) were used for detection. Bands were visualized by enhanced chemiluminescence, and images were taken by FluorChem FC2 Multiimager II (Alpha Innotech, Hessisch Oldendorf, Germany). Intensity of protein bands was calculated with ImageJ software v1.4.3.67.

### 4.6. Real-Time Quantitative PCR

For relative quantification of mRNA, 1 µg of total RNA was subjected to reverse transcription with High-Capacity cDNA Reverse Transcription Kit (Thermo Fisher Scientific). Commercially available TaqMan probes were used with TaqMan^®^ Fast Advanced Master Mix in StepOnePlus Real-Time PCR System (Thermo Fisher Scinetific) as follows: Mm00500330_m1 (Canx); Mm00727012_s1 (Cldn5); Mm01246300_m1 (Trpv1). Calnexin (Canx) was used as endogenous control. Relative expression was calculated by comparative *C*t method.

### 4.7. Data Analysis

Statistical analysis was carried out using Prism v6.0c (GraphPad Software Inc., La Jolla, CA, USA). The Kruskal–Wallis test or one-way ANOVA with Dunnett’s multiple comparisons test were used. All biological experiments were conducted at least in triplicate.

## Supplementary Materials

Supplementary materials can be found at http://www.mdpi.com/1422-0067/20/1/122/s1.

## Abbreviations

BBB	Blood-brain barrier
CCL	capacitance
cEND	brain microvascular endothelial cells
Cldn5	claudin 5
DHM	digital holographic microscopy
ECM	endothelial cell medium
IK	incubation buffer
MTT	3-(4,5-dimethylthiazol-2-yl)-2,5-diphenyltetrazolium bromide
TEER	transendothelial electric resistance
TRITC	tetramethylrhodamine
SIFM	structured illumination fluorescence microscopy
ZO-1	zonula occludens 1

## References

[1] Korel F., Bagdatlioglu N., Balaban M.Ö., Hisil Y. (2002). Ground Red Peppers: Capsaicinoids Content, Scoville Scores, and Discrimination by an Electronic Nose. J. Agric. Food Chem..

[2] Zhang L.L., Liu D.Y., Ma L.Q., Luo Z.D., Cao T.B., Zhong J., Yan Z.C., Wang L.J., Zhao Z.G., Zhu S.J. (2007). Activation of Transient Receptor Potential Vanilloid Type-1 Channel Prevents Adipogenesis and Obesity. Circ. Res..

[3] Smith H., Brooks J.R., Abdel-Salam O.M.E. (2014). Capsaicin-Based Therapies for Pain Control. Capsaicin as a Therapeutic Molecule.

[4] Shiobara T., Usui T., Han J., Isoda H., Nagumo Y. (2013). The Reversible Increase in Tight Junction Permeability Induced by Capsaicin is Mediated Via Cofilin-Actin Cytoskeletal Dynamics and Decreased Level of Occludin. PLoS ONE.

[5] Kaiser M., Goycoolea F.M., Gilliam B. (2014). Vanilloids and Their Effect on Mammalian Biological Barriers. Capsaicin: Food Sources, Medical Uses and Health Implications.

[6] Kaiser M., Pereira S., Pohl L., Ketelhut S., Kemper B., Gorzelanny C., Galla H., Moerschbacher B.M., Goycoolea F.M. (2015). Chitosan Encapsulation Modulates the Effect of Capsaicin on the Tight Junctions of MDCK Cells. Sci. Rep..

[7] Kaiser M., Kirsch B., Hauser H., Schneider D., Seuß-Baum I., Goycoolea F.M. (2015). In Vitro and Sensory Evaluation of Capsaicin-Loaded Nanoformulations. PLoS ONE.

[8] Kaiser M., Chalapala S., Gorzelanny C., Perali R.S., Goycoolea F.M. (2016). The Effect of Capsaicin Derivatives on Tight-Junction Integrity and Permeability of MDCK Cells. J. Pharm. Sci..

[9] Chen Y., Liu L. (2012). Modern Methods for Delivery of Drugs Across the Blood–brain Barrier. Adv. Drug Deliv. Rev..

[10] Beggs S., Liu X.J., Kwan C., Salter M.W. (2010). Peripheral Nerve Injury and TRPV1-Expressing Primary Afferent C-Fibers Cause Opening of the Blood-Brain Barrier. Mol. Pain.

[11] Hu D., Easton A., Fraser P. (2005). TRPV1 Activation Results in Disruption of the Blood-Brain Barrier in the Rat. Br. J. Pharmacol..

[12] Burek M., Salvador E., Förster C.Y. (2012). Generation of an Immortalized Murine Brain Microvascular Endothelial Cell Line as an in Vitro Blood Brain Barrier Model. J. Vis. Exp..

[13] Forster C., Silwedel C., Golenhofen N., Burek M., Kietz S., Mankertz J., Drenckhahn D. (2005). Occludin as Direct Target for Glucocorticoid-Induced Improvement of Blood-Brain Barrier Properties in a Murine in Vitro System. J. Physiol. Lond..

[14] Lee R., Kim J., Kim S.Y., Jang S.M., Lee S.M., Choi I.H., Park S.W., Shin J.S., Yoo K.H. (2012). Capacitance-Based Assay for Real-Time Monitoring of Endocytosis and Cell Viability. Miniat. Chem. Biol..

[15] Wegener J., Keese C.R., Giaever I. (2000). Electric Cell-Substrate Impedance Sensing (ECIS) as a Noninvasive Means to Monitor the Kinetics of Cell Spreading to Artificial Surfaces. Exp. Cell Res..

[16] Nagumo Y., Han J., Bellila A., Isoda H., Tanaka T. (2008). Cofilin Mediates Tight-Junction Opening by Redistributing Actin and Tight-Junction Proteins. Biochem. Biophys. Res. Commun..

[17] Kaiser M., Lankamp F., Goycoolea F.M., Williams P., Phillips G. (2016). Nanoencapsulation of Capsaicin Attenuates the Cytotoxic Effect on Caco-2 Cells. Gums and Stabilisers for the Food Industry 18: Hydrocolloid Functionality for Affordable and Sustainable Global Food Solutions.

[18] Kaiser M., Pohl L., Ketelhut S., Kastl L., Gorzelanny C., Götte M., Schnekenburger J., Goycoolea F.M., Kemper B. (2017). Nanoencapsulated Capsaicin Changes Migration Behavior and Morphology of Madin Darby Canine Kidney Cell Monolayers. PLoS ONE.

[19] Dilling C., Roewer N., Forster C.Y., Burek M. (2017). Multiple Protocadherins are Expressed in Brain Microvascular Endothelial Cells and might Play a Role in Tight Junction Protein Regulation. J. Cerebr. Blood Flow Metabol..

