# Supplementary material for: The Influence of Capsaicin on the Integrity of Microvascular Endothelial Cell Monolayers
Source: Int J Mol Sci. 2018 Dec 30;20(1):122. doi: 10.3390/ijms20010122 (PMC6337111; doi:10.3390/ijms20010122)
Supplement: Supplementary file 1 [file ijms-20-00122-s001.pdf]

International Journal of
Molecular Sciences

MDPI

Supplementary: The Influence of Capsaicin on the Integrity of Microvascular Endothelial Cell Monolayers

Mathias Kaiser [1,2,†], Malgorzata Burek [3†], Stefan Britz [2], Frauke Lankamp [2], Steffi Ketelhut [4], Björn Kemper [4], Carola Förster [3], Christian Gorzelanny [5], Francisco M. Goycoolea [2,6,*]

[1] Max Delbrück Center for Molecular Medicine, Robert-Rössle-Straße 10, Berlin 13125, Germany; Mathias.Kaiser@mdc-berlin.de (M.K.)
[2] Institute of Plant Biology and Biotechnology (IBBP), Westfälische Wilhelms-Universität Münster, Schlossgarten 3, Münster 48149, Germany; stefan.bulla@gmx.at (S.B.), f_lank01@uni-muenster.de (F.L.)
[3] Deparment of Anaesthesia and Critical Care, University of Würzburg, Oberdürrbacher Straße 6, Würzburg 97080, Germany; Burek_M@ukw.de (M.B.); foerster_c@ukw.de (C.F.)
[4] Biomedical Technology Center of the Medical Faculty, Westfälische Wilhelms-Universität Münster Mendelstraße 17, Münster 48149, Germany; ketelhut@uni-muenster.de (S.K.); bkemper@uni-muenster.de (B.K.)
[5] Department of Dermatology and Venerology, University Medical Center Hamburg-Eppendorf, Hamburg 20246, Germany; c.gorzelanny@uke.de
[6] School of Food Science and Nutrition, University of Leeds, Leeds LS2 9JT, UK
* Correspondence: F.M.Goycoolea@leeds.ac.uk; Tel.: +44(0)113 343 1412
† These authors contributed equally to this work.

Digital holographic microscopy (DHM)

For imaging studies with DHM, cells were seeded in Petri dishes with glass lid (ibidi µ-Dish with glass lid, ibidi GmbH, Munich, Germany) in supplemented ECM at a density of 2.1 x 105 cells/dish and were allowed to attach overnight. The following day the medium was replaced by ECM lacking fetal calf serum but containing 50 µM capsaicin in 20 mM HEPES buffer. DHM imaging was carried out using an inverted microscope (iMIC, Till Photonics, Gräfelfing, Germany) with an attached DHM module [1] with an incubator set at 37°C. The coherent light source was a Nd:YAG laser (Compass 315 M-100, Coherent, Lübeck, Germany, λ =532 nm). The digital holograms of single confluent cell layers were recorded continuously every 9 min using a 20x microscope lens (Zeiss LD Acroplan 20x/0.4 Korr). Quantitative phase images were reconstructed from the digitally-captured holograms as previously described [2, 3]. Three independent measurements were taken in each experiment.

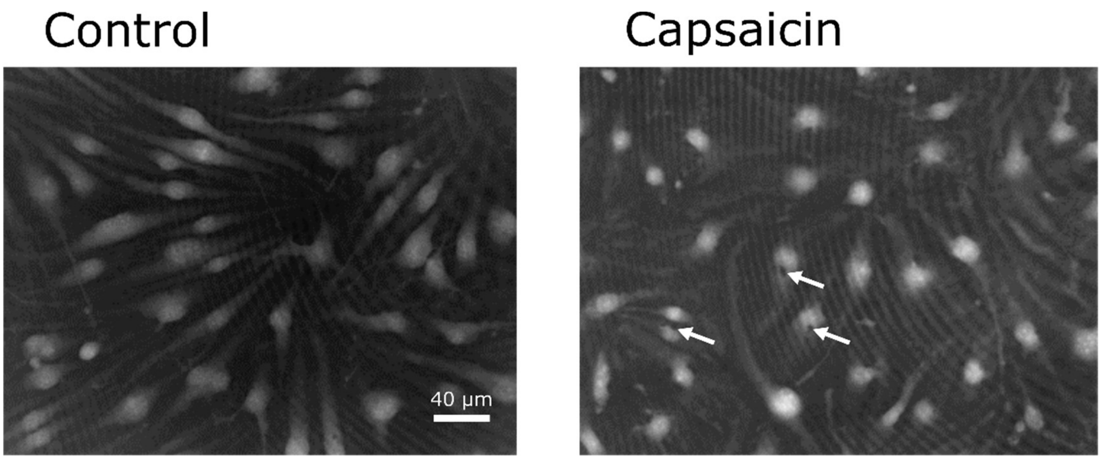

Figure S1. Representative DHM quantitative phase images of cEND cell monolayers. Cells remained untreated or were treated with 50 μM capsaicin for 16 h. Arrows indicate morphological changes.

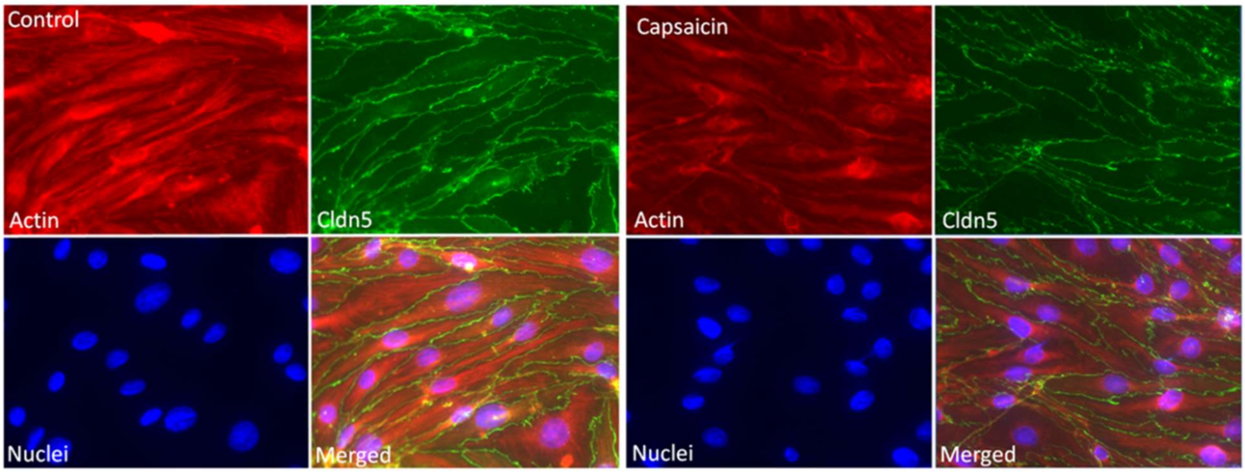

Figure S2. SIFM images of primary mouse brain microvascular endothelial cells treated with capsaicin. Cells remained untreated or were treated with 100 μM capsaicin for 12 h. Nuclei were stained with DAPI (blue), claudin 5 was stained using specific antibodies (green) and actin was stained with TRITC-phalloidin (red), magnification 400x.

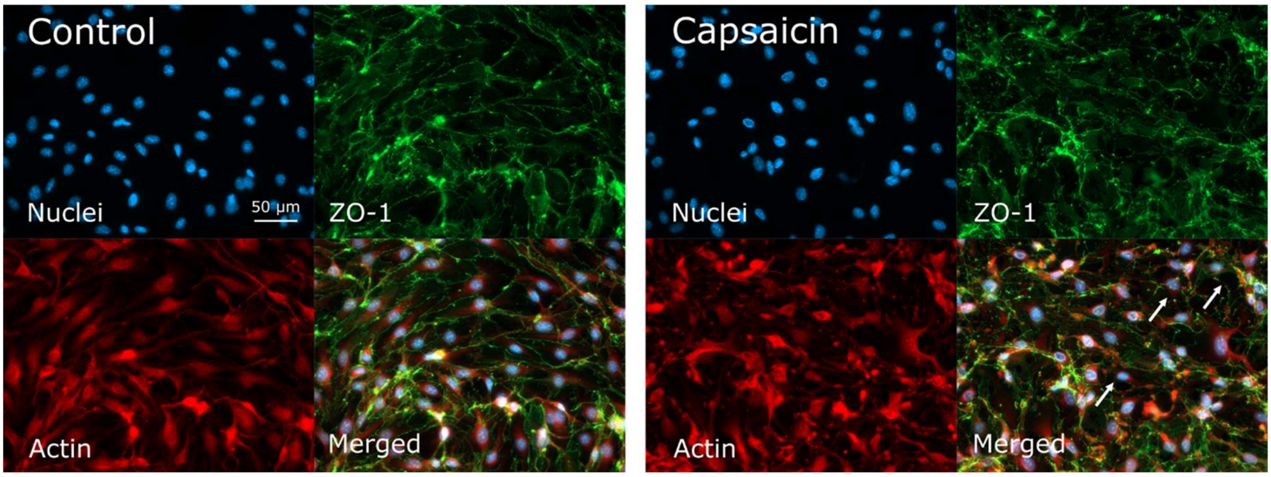

Figure S3. SIFM images of cEND cells treated with capsaicin. Cells remained untreated or were treated with 100 μM capsaicin for 12 h. Nuclei were stained with DAPI (blue), zonula occludens 1 (ZO-1) was stained using specific antibodies (green) and actin was stained with TRITC-phalloidin (red). Arrows indicate changes in protein localization.

Literature Cited

1. Kemper B, Carl D, Höink A, Von Bally G, Bredebusch I, Schnekenburger J 2006. Modular digital holographic microscopy system for marker free quantitative phase contrast imaging of living cells. Proc. SPIE 6191, 61910T.

2. Carl D, Kemper B, Wernicke G, Von Bally G 2004. Parameter-optimized digital holographic microscope for high-resolution living-cell analysis. Appl Opt 43:6536-6544.

3. Kemper B, Carl D, Schnekenburger J, Bredebusch I, Schäfer M, Domschke W, Von Bally G 2006. Investigation of living pancreas tumor cells by digital holographic microscopy. J Biomed Opt 11, 034005.
